# Constraints, mechanisms, and strategies for industry-education integration in vocational education: An empirical study

**DOI:** 10.1371/journal.pone.0339158

**Published:** 2025-12-18

**Authors:** Bo Yu, Ruipu Li, Siyuan Zhang, Peng Mao, Chun Xu, Jing Shi, Gang Wu

**Affiliations:** 1 School of Construction Engineering, Shenzhen Polytechnic University, Shenzhen, China; 2 Quality Assurance Center, Shenzhen Polytechnic University, Shenzhen, China; 3 School of Construction Engineering, Guangzhou Vocational University of Science and Technology, Guangzhou, China; 4 College of Civil Engineering, Nanjing Forestry University, Nanjing, China; Nanjing Audit University, CHINA

## Abstract

Identifying the constraints hindering industry-education integration (IEI) in vocational education is a critical prerequisite for promoting its high-quality development. However, existing research has yet to establish a systematic solution to this core issue. Therefore, this study takes Shenzhen, China, as a case study and adopts an empirical research approach to construct an analytical framework of “factor identification-mechanism analysis-strategy formulation,” aiming to thoroughly investigate the key constraints on IEI development in vocational education. First, based on a literature review and questionnaire survey, the study identifies 12 critical constraining factors of IEI. Subsequently, through expert interviews and Interpretative Structural Modelling (ISM), it reveals the underlying mechanisms of these constraints, classifying the 12 factors into five hierarchical levels. These factors form 16 constraint pathways, with inadequate policies, weak foundations for school-enterprise collaboration, insufficient institutional commitment, and ineffective communication mechanisms identified as critical bottom-level factors. These indirectly influence intermediate factors such as hardware/software conditions, alignment of talent cultivation philosophies, education-market demand matching, collaboration channel efficiency, and stakeholder interest fulfillment. Consequently, they diminish corporate engagement enthusiasm, blur responsibility-rights boundaries between stakeholders, and dampen faculty participation motivation, ultimately impeding IEI progress. Finally, the study proposes targeted countermeasures, emphasizing tripartite collaboration among the government, vocational institutions, and enterprises. Key strategies include strengthening legal safeguards, consolidating industry-education collaboration foundations, and proactively fulfilling educational responsibilities. The findings resolve the dilemmas constraining IEI development, providing policymakers with evidence-based references and offering practical guidance for deeper collaboration between vocational institutions and enterprises. This study holds significant theoretical and practical value for advancing high-quality vocational education.

## 1. Introduction

With the rapid development of globalization and technology, industrial structure and economic forms are undergoing profound changes [1,2]. Traditional education models can no longer meet the demand for highly skilled talent in modern society, especially against the backdrop of economic transformation and upgrading, making vocational education increasingly important [3,4]. In China, IEI has been elevated to a national strategic height: it is explicitly positioned as a core measure in the “National Vocational Education Reform Implementation Plan” (2019) to address the mismatch between talent supply and industrial demand, and further emphasized in the “14th Five-Year Plan for Vocational Education Development” (2021–2025) as a key pathway to build a skills-based society and support high-quality economic development. Against this macro backdrop, deepening IEI has become not only a requirement for vocational education reform but also a critical support for China’s industrial upgrading and sustainable economic growth. As an important avenue for cultivating skilled technical talent, vocational education plays a crucial role in supporting social and economic development [5,6]. Industry-education integration (IEI), as a key strategic measure to promote vocational education, refers to the deep cooperation between education and industry, aiming to align educational content closely with industry needs to enhance educational quality and ensure precise talent development [7]. Currently, IEI has become a comprehensive institutional arrangement for vocational education reform and talent resource development.

However, since IEI is a complex system project involving multiple stakeholders (such as the government, universities, and enterprises), its development faces numerous challenges [8]. These include insufficient policy support, an unreasonable management system, underdeveloped IEI platforms, imperfect resource allocation mechanisms, and low levels of talent sharing between universities and enterprises [9–11]. Under these constraints, the role of the government and social organizations in the integration process of vocational education is limited, and the channels for university-enterprise collaboration are not smooth enough. Furthermore, the conflict between the public welfare nature of universities and the profit-oriented demands of enterprises often leads to disputes over property rights, profit distribution, and responsibility allocation, which severely hinders the effective implementation of vocational education and IEI [12–14]. Therefore, it is necessary to explore the reasons restricting the development of vocational education and IEI at the current stage and propose targeted and effective countermeasures to promote the high-quality development of IEI in vocational education.

Current research on IEI in vocational education has garnered significant academic attention. A systematic review of existing studies reveals that scholarly focus has primarily centered on integration models, evaluation systems, talent cultivation, and influencing factors. Regarding integration models, extant research has examined Germany’s “dual system,” America’s “cooperative education,” and Australia’s “training package” approaches [15–18]. Some scholars have attempted to transplant these models to other contexts [19,20]. For instance, Hong investigated the applicability of Germany’s dual system in China, finding direct adoption problematic without contextual adaptation and innovation [21]. Consequently, researchers have proposed context-specific integration models accounting for regional and industrial particularities. Notable examples include Li et al.’s framework for China’s water conservancy sector, featuring physical hybrid, biological grafting, chemical compounding, and ecological symbiosis models, demonstrating the necessity of localized model development [22].

Regarding evaluation systems, existing research indicates that developed countries generally emphasize the assessment of IEI, albeit with varying evaluation approaches and requirements across nations [7]. Germany employs a dual evaluation mechanism where vocational colleges develop quality rating scales combining internal and external assessments [23]. The United States prioritizes effective articulation between vocational education and industrial sectors, mandating state-level development of evaluation plans and performance indicators [24]. In Japan, quality evaluation constitutes a statutory obligation for vocational institutions, implemented through combined self-evaluation and external review processes [25]. Furthermore, scholars have developed diverse evaluation frameworks using distinct methodologies: Chen et al. established an urban IEI index system by applying modified fuzzy linguistic approaches based on the Context-Input-Process-Product model [26]; Gong constructed a performance evaluation model grounded in coupling coordination theory, incorporating the Structure-Conduct-Performance paradigm from industrial organization theory [8]; while Qi and Feng proposed an effectiveness evaluation model utilizing backpropagation neural networks [27].

Regarding talent cultivation, existing research has primarily focused on addressing the question of “how to develop high-quality vocational talents through IEI.” These studies have proposed recommendations and strategies to enhance talent cultivation quality, mainly from perspectives of curriculum systems, teaching models, and instructional methods [28]. For instance, Laine et al. argued that vocational institutions should leverage their unique strengths to establish talent cultivation curriculum systems, continuously enrich teaching models, and adopt diversified pedagogical approaches within IEI [29]. Whittle and Hutchinson emphasized the need to prioritize the coordination between social development and vocational education advancement, suggesting that vocational colleges should design hierarchical curriculum systems, implement reasonable assessment methods for internship programs, and balance student engagement with corporate interests to stimulate enterprise motivation and ensure talent cultivation quality [30]. Furthermore, discipline-specific investigations have been conducted, such as Ren et al.’s exploration of objectives and models for geology talent cultivation, which identified directions for geological education reform and proposed improvements through new training programs and innovations in teaching content and methodologies [31].

Regarding influencing factors, scholars have investigated key determinants affecting the implementation effectiveness of IEI. For instance, Pan and Zhang contend that the ultimate outcomes of vocational education integration depend not on isolated factors but rather on the synergistic interplay of multiple elements, including institutional capacity of vocational colleges, government intervention, and corporate engagement [32]. Thune emphasizes that successful integration hinges on four critical dimensions: goal-aligned partnerships, robust collaboration mechanisms, conflict-resolution communication competencies, and sustainable funding [33]. Furthermore, given that integration should be grounded in stakeholder needs, some studies adopt specific stakeholder perspectives to examine participation willingness and its determinants. A case in point is Zheng et al., who surveyed 184 hydrogen energy enterprises to analyze how resource benefits, economic costs, and policy subsidies influence corporate engagement willingness [34]. Their findings reveal that universities’ provision of high-quality resources significantly enhances corporate participation propensity.

Current research has effectively promoted the development of IEI in vocational education to some extent, but there are still certain limitations. First, many scholars primarily focus on specific issues that need to be addressed during the implementation of IEI, such as which model to adopt or what kind of evaluation system to construct. However, few scholars have conducted systematic and in-depth research on the difficulties existing in the development of IEI, resulting in a lack of corresponding solutions and measures. Second, although some scholars have conducted research on the challenges in the development of IEI in vocational education, these studies mainly propose countermeasures from a macro perspective based on specific cases. Due to the lack of in-depth analysis of the underlying causes restricting the development of IEI, the proposed countermeasures may deviate from actual situations, potentially leading to counterproductive outcomes. Therefore, it is necessary to explore the factors and mechanisms restricting the development of IEI in vocational education in greater depth, in order to propose targeted countermeasures that will help overcome the current challenges in the development of IEI.

To address this identified research gap, this study selects Shenzhen, China as an empirical case to explore the constraining factors and underlying mechanisms hindering the integration of vocational education and industry, while proposing effective countermeasures. Shenzhen serves as an exemplary research context due to its dual identity as both a global manufacturing hub and a national pilot city for educational reform. As a national pilot, Shenzhen has formulated a multi-tiered policy framework for vocational education IEI, anchored by documents such as Shenzhen Three-Year Action Plan for High-Quality Development of Technical and Vocational Education (2023–2025), the draft Shenzhen Special Economic Zone Vocational Education Regulations, Shenzhen Work Plan for Improving the Lifelong Vocational Skills Training System, and Shenzhen Measures for Vocational Skills Training Subsidies. Specifically, the Three-Year Action Plan explicitly mandates the construction of 10 industry colleges with benchmark enterprises and 20 model industry-education integration training bases by 2025, while stipulating that enterprises participating in collaboration are eligible for fiscal, financial, and tax incentives. The draft Vocational Education Regulations further clarifies the legal obligations and incentive mechanisms for enterprise participation in IEI, and the Training Subsidies Measures provides tangible financial support for enterprises engaged in vocational skills training, forming a policy system covering institutional guarantees, resource input, and benefit incentives. In addition, Shenzhen hosts a concentration of high-tech industries (e.g., Huawei, Tencent) and vocational institutions, presenting a microcosm of both tensions and synergies in workforce supply-demand dynamics. The city’s rapid industrial upgrading and explicit policy mandates necessitate close industry-education collaboration [35]. However, persistent challenges, including curriculum misalignment with Industry 4.0 skill requirements and uneven enterprise participation – mirror systemic issues commonly faced by emerging economies. Consequently, using Shenzhen as an empirical case offers transferable insights for other similar emerging economies that aim to enhance vocational education-industry integration.

To achieve the research objectives, this study first employed a literature review and questionnaire surveys to identify the constraining factors affecting the development of IEI in vocational education. Then, by using expert interviews and Interpretative Structural Modelling (ISM), the study elucidated the mechanisms that restrict the development of IEI and reveals specific pathways of constraint. Finally, response strategies to promote the integrated development of industry-education collaboration in vocational education were proposed from three key stakeholders: the government, vocational institutions, and enterprises.

## 2. Research process and methods

**Fig 1:**
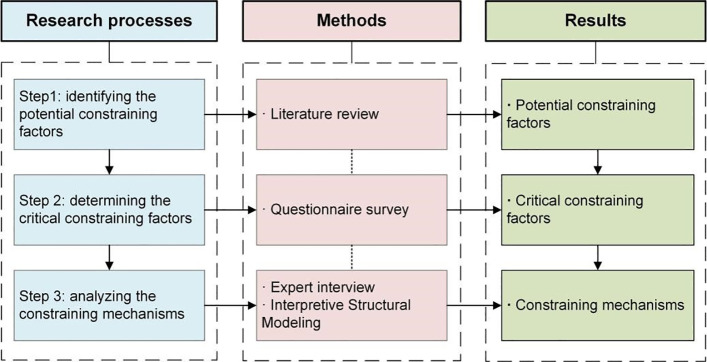
The research process and methods.

Since existing research has not yet systematically explored the constraining factors and mechanisms of IEI, this study draws upon research methodologies concerning influencing factors and mechanisms from other related fields to fill this gap [36–38]. Through synthesis, their research processes can be categorized into three distinct phases: (1) identifying the potential constraining factors, (2) determining the critical constraining factors, and (3) analyzing the constraining mechanisms. Consequently, this study adopts the same three-phase research framework, wherein the research methods used at each stage, along with the corresponding outcomes, are illustrated in Fig 1.

### 2.1. Identifying the potential constraining factors

The literature review is widely acknowledged as an effective method for identifying potential factors and has been extensively utilized in contemporary research [37,39–41]. This study also employs a literature review to identify the potential factors that constrain the development of IEI in vocational education. Specifically, the study draws on research findings related to IEI, sourced from major databases such as ScienceDirect and Web of Science, to establish a comprehensive foundation for the review. Initially, relevant literature was collected using keywords such as “industry-education integration,” “integration of industry and education,” “school-enterprise cooperation,” and “school-enterprise collaboration.” The selected literature was systematically reviewed, resulting in the identification of 13 potential factors that hinder the development of IEI. These factors include: the completeness of policies, the willingness of enterprises to cooperate, the alignment of talent cultivation quality, the level of attention given by universities, the degree of teacher involvement, the extent of student engagement, the adequacy of hardware and software facilities, the differences in talent cultivation philosophies, the achievement of school-enterprise mutual interests, the clarity of school-enterprise responsibilities and rights, the effectiveness of school-enterprise communication, the closeness of prior university-enterprise collaboration, and the fluidity of school-enterprise cooperation channels. The definitions and sources of these potential constraining factors are provided in Table 1.

**Table 1:** Definitions and sources of potential constraining factors.

No.	Potential constraining factors	Definitions	Sources
1	The completeness of policies	The degree of perfection in policies, regulations, standards, and norms related to IEI.	[34,42,43]
2	The willingness of enterprises to cooperate	Enterprises’ willingness to engage in IEI with higher education institutions.	[13,34]
3	The alignment of talent cultivation quality	The degree of alignment between the quality of talent cultivation in higher education institutions and enterprise employment requirements.	[42,44]
4	The level of attention given by universities	The degree of emphasis higher education institutions place on implementing IEI.	[32]
5	The degree of teacher involvement	The willingness and enthusiasm of faculty members to participate in IEI.	[34]
6	The extent of student engagement	The willingness and enthusiasm of students to participate in IEI.	[27,30]
7	The adequacy of hardware and software facilities	The degree of perfection in software/hardware facilities (e.g., faculty, training venues, equipment) during IEI.	[7,14]
8	The differences in talent cultivation philosophies	The degree of difference between higher education institutions and enterprises regarding vocational education talent cultivation concepts.	[45]
9	The achievement of school-enterprise mutual interests	The degree to which the interests and expectations of higher education institutions and enterprises are achieved through IEI.	[13,34,46]
10	The clarity of school-enterprise responsibilities and rights	The degree of clarity in the rights, responsibilities, and obligations of higher education institutions and enterprises in IEI.	[12,13,24]
11	The effectiveness of school-enterprise communication	The degree of effectiveness in communication and interaction between higher education institutions and enterprises during IEI.	[42,47,48]
12	The closeness of prior university-enterprise collaboration	The degree of closeness in collaboration between higher education institutions and enterprises prior to implementing IEI.	[49]
13	The fluidity of school-enterprise cooperation channels	The degree of smoothness in collaboration channels between higher education institutions and enterprises.	[49,50]

### 2.2. Determining the critical constraining factors

Determining the critical constraining factors is essential for unraveling the underlying mechanisms that impede the development of IEI. A questionnaire survey, as a widely used method for identifying critical factors in contemporary research [37,51,52], was consequently adopted in this study. Specifically, the survey focuses on stakeholders who are closely involved in the integration of vocational education and industry, including government agencies, vocational educational institutions, and enterprises, all based in Shenzhen, China. The questionnaire comprises two sections. The first section collects fundamental demographic data regarding the respondents, including gender, age, education level, profession, years of work experience, and their understanding their comprehension of IEI within the context of vocational education. This section aims to ensure that the selection of respondents is both scientifically valid and representative. The second section comprises items designed to measure the degree of constraint imposed by the identified potential constraining factors, as detailed in Table 2. A total of 13 items are included, each corresponding to one of the 13 previously identified potential constraining factors. Furthermore, this study utilizes a five-point Likert scale to assess the level of constraint associated with each factor. Respondents are asked to rate each item on a scale from “1” to “5,” corresponding to “Strongly Disagree,” “Disagree,” “Neutral,” “Agree,” and “Strongly Agree,” respectively. The survey was conducted from October 2023 to February 2024, utilizing both online and offline methods for distribution and data collection. In total, 624 questionnaires were collected, with 534 valid responses.

**Table 2:** Measurement items for potential constraining factors.

No.	Potential constraining factors	Measurement items
1	The completeness of policies	I believe that the inadequacy of policies related to IEI will constrain its development.
2	The willingness of enterprises to cooperate	I believe that the lack of strong willingness from enterprises to engage in IEI will constrain its development.
3	The alignment of talent cultivation quality	I believe that the failure of talent cultivation quality to meet the employment requirements of enterprises will constrain the development of IEI.
4	The level of attention given by universities	I believe that the insufficient attention given by universities to IEI will constrain its development.
5	The degree of teacher involvement	I believe that the low level of teacher participation in IEI will constrain its development.
6	The extent of student engagement	I believe that the low level of student participation in IEI will constrain its development.
7	The adequacy of hardware and software facilities	I believe that the lack of hardware and software facilities, such as faculty, training sites, and equipment, will constrain the development of IEI.
8	The differences in talent cultivation philosophies	I believe that the differences in talent cultivation philosophies between universities and enterprises will constrain the development of IEI.
9	The achievement of school-enterprise mutual interests	I believe that the failure to meet mutual expectations regarding interests between universities and enterprises will constrain the development of IEI.
10	The clarity of school-enterprise responsibilities and rights	I believe that the lack of clarity regarding the responsibilities and rights of universities and enterprises will constrain the development of IEI.
11	The effectiveness of school-enterprise communication	I believe that the lack of effective communication between universities and enterprises will constrain the development of IEI.
12	The closeness of prior university-enterprise collaboration	I believe that the weak foundation of early-stage cooperation between universities and enterprises will constrain the development of IEI.
13	The fluidity of school-enterprise cooperation channels	I believe that the insufficient smoothness of cooperation channels between universities and enterprises will constrain the development of IEI.

To ensure the accuracy of the survey results and drawing upon existing research findings [38], this study identifies potential constraining factors as critical constraining factors if they are rated at 4 or higher by more than 80% of respondents. The survey results are shown in S1 Text. The findings reveal that, with the exception of the factor “The extent of student engagement,” all other 12 potential constraining factors were rated 4 or higher by 80% of the respondents. Therefore, these 12 factors are identified as critical constraining factors for the development of IEI in vocational education.

### 2.3. Analyzing the constraining mechanisms

The analysis of constraining mechanisms constitutes the most critical research phase, as it is fundamental for formulating targeted strategies to effectively promote IEI in vocational education. This study employed a comprehensive methodological approach combining expert interviews with ISM to systematically examine the interrelationships among constraining factors and identify key constraining pathways. This dual-method approach enables us to elucidate the underlying mechanisms that constrain the development of IEI in vocational education.

#### 2.3.1. Expert interview.

This study employed expert interviews to determine the interrelationships among various constraining factors. To ensure the validity of the investigation and the accuracy of results, this study invited 30 experts from government agencies, vocational institutions, and enterprises who possess profound insights into IEI in vocational education and have substantial research and practical experience in this field. The sample reached theoretical saturation when no new findings emerged after interviewing the 21st participant and subsequent interviewees. The criteria for judging theoretical saturation in this study were defined based on three core principles, consistent with established qualitative research standards [37,38]:

(1) *No new insights on factor interrelationships.* After the 21st interview, subsequent experts did not propose additional types of influence relationships between the 12 constraining factors (e.g., no new direct/indirect impact pathways beyond those identified in earlier interviews);
(2) *Consistency in key judgments.* The degree of agreement on critical factor relationships (e.g., the influence of “policy completeness” on “school attention level”) stabilized at over 80% (matching the study’s pre-established inter-rater agreement threshold) and no contradictory judgments on core mechanisms emerged;
(3) *Sufficiency of explanatory logic.* All rationales for factor relationships provided by later interviewees were repetitions of arguments from earlier participants, with no new theoretical or practical justifications for constraint mechanisms added.

The interview process followed a structured protocol: First, participants were briefed on the research background and objectives, followed by an introduction to the 12 pre-identified constraining factors. Subsequently, experts completed a standardized documentation to assess the interrelationships among these factors. Finally, based on their documented responses, in-depth interviews were conducted to verify the identified constraining factors and explore the underlying rationales for these relationships.

The face-to-face interviews were conducted between March and May 2024. S2 Text presents the demographic characteristics of the interviewees. Recognizing potential inter-rater discrepancies, this study established an 80% agreement threshold – a widely adopted benchmark in existing studies – to validate the findings [38]. Only relationships endorsed by over 80% of respondents were considered statistically significant, thereby ensuring methodological rigor and result reliability.

#### 2.3.2. ISM.

**Fig 2:**
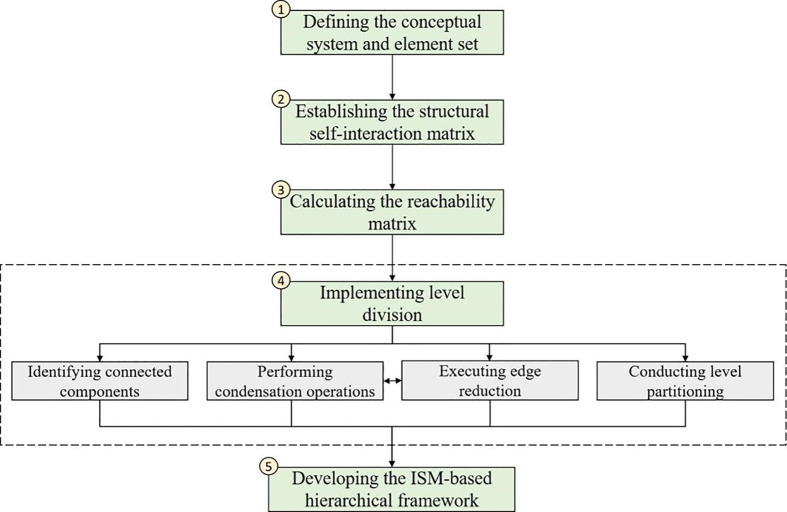
Implementation steps of ISM.

ISM is a systematic analytical approach that primarily utilizes expert knowledge to examine the interrelationships among various elements within a system [53]. By employing directed graphs, matrices, and computer programming techniques, ISM converts complex and ambiguous relationships among system elements into a clear, intuitive, and hierarchical structural model, thereby enhancing its readability and reference value [54]. This method is particularly suitable for investigating multi-factor interaction mechanisms [55,56]. Therefore, this study employs ISM to analyze the constraining mechanisms affecting IEI development in vocational education. With reference to existing research, the implementation adheres to a five-step procedure illustrated in Fig 2: (1) defining the conceptual system and its corresponding element set, (2) establishing the structural self-interaction matrix, (3) computing the reachability matrix, (4) implementing level partition, and (5) establishing the ISM-based hierarchical framework.

(1) *Defining the conceptual system and element set.* The conceptual system refers to the research problem and system boundaries that will be addressed using ISM, whereas the element set consists of specific influencing factors under investigation. Aligned with the research objectives, this study defines the conceptual system as the constraining mechanisms for the development of IEI in vocational education, which includes both encompassing constraining factors and their corresponding pathways. The element set consists of the 12 constraining factors identified in Section 2.2.
(2) *Establishing the structural self-interaction matrix.* The structural self-interaction matrix quantifies the pairwise influence relationships among system elements. In this study, the matrix was constructed based on expert interview results outlined in Section 2.3.1.
(3) *Calculating the reachability matrix.* The reachability matrix is used to explicitly delineate the presence of influence relationships among system elements, encompassing both direct and indirect effects. This matrix is derived through Boolean algebraic operations performed on the structural self-interaction matrix, with its computational implementation realized through MATLAB programming.
(4) *Implementing level division.* Hierarchical partitioning was conducted by using the reachability matrix to determine the tier of each factor within the system. The computational procedure consisted of four main steps:
  (a) Identifying connected components: This task aims to group elements that have direct or indirect influence relationships into independent sub-systems. It helps eliminate isolated elements (if any) that have no connection to the core constraint system, ensuring the subsequent analysis focuses only on mutually related factors that truly affect IEI development.
  (b) Performing condensation operations: This step simplifies the complex network of element relationships by merging elements within the same connected component into a single “condensed node” if they have identical reachability and antecedent sets. Its purpose is to reduce redundant calculations and clarify the hierarchical structure by removing duplicate or highly overlapping element relationships.
  (c) Executing edge reduction: This involves removing transitive edges (i.e., indirect influence relationships that can be inferred through intermediate elements) from the network. It streamlines the relationship structure, avoiding over-complication of the model and making the direct causal links between elements more prominent.
  (d) Conducting level partitioning. Specifically, for each factor, the reachability set, antecedent set, and intersection set were computed from the reachability matrix, which facilitated the assignment of tiers based on predefined rules (i.e., an element is assigned to a level if its reachability set intersects with its antecedent set exactly at the reachability set itself).
(5) *Developing the ISM-based hierarchical framework.* The final hierarchical framework was established by integrating level division results with the reachability matrix, thereby ensuring a systematic and comprehensive structure. Represented through nodes (denoting elements) and directed edges (indicating influence relationships), the framework adheres to the principle of simplification by removing redundant transitive paths. This visualization enhances model clarity and facilitates intuitive understanding of systemic structures.

## 3. Results and discussion

### 3.1. The structural self-interaction matrix

Based on the interview results, the structural self-interaction matrix established in this study is shown in Table 3. “V” indicates that the constraint *S*_*i*_ in the row had a direct impact on the constraint *S*_*j*_ in the corresponding column, such as “*S*_*1*_ (The completeness of policies)” had a direct impact on “*S*_*4*_ (The level of attention given by universities)”; “A” means that the constraint *S*_*j*_ in the column had a direct impact on the constraint *S*_*i*_ in the corresponding row, such as “*S*_*3*_ (The alignment of talent cultivation quality)” had a direct impact on “*S*_*2*_ (The willingness of enterprises to cooperate)”; and “O” denotes that there was no direct impact between the constraint *S*_*i*_ in the row and the constraint *S*_*j*_ in the corresponding column, such as “*S*_*1*_ (The completeness of policies)” and “*S*_*11*_ (The closeness of prior university-enterprise collaboration)”.

**Table 3:** The structural self-interaction matrix.

Constraint factors^a^	*S*_*12*_	*S*_*11*_	*S*_*10*_	*S*_*9*_	*S*_*8*_	*S*_*7*_	*S*_*6*_	*S*_*5*_	*S*_*4*_	*S*_*3*_	*S*_*2*_
*S*_*1*_	*O*	*O*	*O*	*V*	*O*	*O*	*O*	*O*	*V*	*O*	*O*
*S*_*2*_	*A*	*A*	*A*	*O*	*A*	*A*	*O*	*O*	*O*	*A*	
*S*_*3*_	*O*	*A*	*A*	*O*	*O*	*A*	*A*	*O*	*O*		
*S*_*4*_	*V*	*O*	*O*	*O*	*O*	*O*	*V*	*V*			
*S*_*5*_	*O*	*A*	*A*	*O*	*O*	*O*	*O*				
*S*_*6*_	*O*	*O*	*O*	*O*	*O*	*O*					
*S*_*7*_	*O*	*O*	*A*	*O*	*O*						
*S*_*8*_	*O*	*O*	*A*	*O*							
*S*_*9*_	*O*	*O*	*A*								
*S*_*10*_	*V*	*A*									
*S*_*11*_	*V*										

^a^*S*_*1*_ (The completeness of policies), *S*_*2*_ (The willingness of enterprises to cooperate), *S*_*3*_ (The alignment of talent cultivation quality), *S*_*4*_ (The level of attention given by universities), *S*_*5*_ (The degree of teacher involvement), *S*_*6*_ (The adequacy of hardware and software facilities), *S*_*7*_ (The differences in talent cultivation philosophies), *S*_*8*_ (The achievement of school-enterprise mutual interests), *S*_*9*_ (The clarity of school-enterprise responsibilities and rights), *S*_*10*_ (The effectiveness of school-enterprise communication), *S*_*11*_ (The closeness of prior university-enterprise collaboration), *S*_*12*_ (The fluidity of school-enterprise cooperation channels).

### 3.2. The reachability matrix

The reachability matrix, calculated based on the structural self-interaction matrix in this study, is shown in Table 4. In the reachability matrix, “1” indicates that the constraint *S*_*i*_ in the row directly or indirectly influences the constraint *S*_*j*_ in the corresponding column, such as “*S*_*1*_ (The completeness of policies)” had affected “*S*_*2*_ (The willingness of enterprises to cooperate)”, “*S*_*3*_ (The alignment of talent cultivation quality)”, “*S*_*4*_ (The level of attention given by universities)”, “*S*_*5*_ (The degree of teacher involvement)”, “*S*_*6*_ (The adequacy of hardware and software facilities)”, “*S*_*9*_ (The clarity of school-enterprise responsibilities and rights)”, and “*S*_*12*_ (The fluidity of school-enterprise cooperation channels)”; “0” indicates that the constraint *S*_*i*_ in the row does not directly or indirectly influence the constraint *S*_*j*_ in the corresponding column, such as “*S*_*1*_ (The completeness of policies)” had not affected “*S*_*7*_ (The differences in talent cultivation philosophies)”, “*S*_*8*_ (The achievement of school-enterprise mutual interests)”, “*S*_*10*_ (The effectiveness of school-enterprise communication)”, and “*S*_*11*_ (The closeness of prior university-enterprise collaboration)”.

**Table 4:** The reachability matrix.

Constraint factors	*S*_*1*_	*S*_*2*_	*S*_*3*_	*S*_*4*_	*S*_*5*_	*S*_*6*_	*S*_*7*_	*S*_*8*_	*S*_*9*_	*S*_*10*_	*S*_*11*_	*S*_*12*_	Driving power^a^
*S*_*1*_	1	1	1	1	1	1	0	0	1	0	0	1	8
*S*_*2*_	0	1	0	0	0	0	0	0	0	0	0	0	1
*S*_*3*_	0	1	1	0	0	0	0	0	0	0	0	0	2
*S*_*4*_	0	1	1	1	1	1	0	0	0	0	0	1	6
*S*_*5*_	0	0	0	0	1	0	0	0	0	0	0	0	1
*S*_*6*_	0	1	1	0	0	1	0	0	0	0	0	0	3
*S*_*7*_	0	1	1	0	0	0	1	0	0	0	0	0	3
*S*_*8*_	0	1	0	0	0	0	0	1	0	0	0	0	2
*S*_*9*_	0	0	0	0	0	0	0	0	1	0	0	0	1
*S*_*10*_	0	1	1	0	1	0	1	1	1	1	0	1	8
*S*_*11*_	0	1	1	0	1	0	1	1	1	1	1	1	9
*S*_*12*_	0	1	0	0	0	0	0	0	0	0	0	1	2
Dependence power^b^	1	10	7	2	5	3	3	3	4	2	1	5	46

^a^Driving force refers to the extent to which a constraint influences other constraints. The greater the driving force, the more constraints it influences.

^b^Dependence power refers to the extent to which a constraint is influenced by other constraints. The greater the dependence power, the more constraints it is influenced by.

### 3.3. The level partition

Based on the reachability matrix, this study systematically calculated the reachability, previous, and intersection sets of each constraint, and determined the level partition of each constraint according to the calculation rule *L*_*1*_*={S*_*i*_*|R(S*_*i*_*)∩Q(S*_*i*_*)=R(S*_*i*_*), i* = 0,1,2,…, *k},* as shown in Table 5. The results indicated that the 12 constraints are divided into five levels. Specifically, the constraints “S_2_ (The willingness of enterprises to cooperate),” “S_5_ (The degree of teacher involvement),” and “S_9_ (The clarity of school-enterprise responsibilities and rights)” were classified into *Level I*; “S_3_ (The alignment of talent cultivation quality),” “S_8_ (The achievement of school-enterprise mutual interests),” and “S_12_ (The fluidity of school-enterprise cooperation channels)” were located at *Level II*; “S_6_ (The adequacy of hardware and software facilities)” and “S_7_ (The differences in talent cultivation philosophies)” belong to *Level III*; “S_4_ (The level of attention given by universities)” and “S_10_ (The effectiveness of school-enterprise communication)” were at *Level IV*; “S_1_ (The completeness of policies)” and “S_11_ (The closeness of prior university-enterprise collaboration)” were placed in *Level V*.

**Table 5:** The level partition (iterations 1 to 5 combined).

Constraint factors	Reachability set	Antecedent set	Intersection set	Level
*S*_*1*_	*S*_*1*_ *, S*_*2*_ *, S*_*3*_ *, S*_*4*_ *, S*_*5*_ *, S*_*6*_ *, S*_*9*_ *, S*_*12*_	*S*_*1*_	*S*_*1*_	*V*
*S*_*2*_	*S*_*2*_	*S*_*1*_ *, S*_*2*_ *, S*_*3*_ *, S*_*4*_ *, S*_*6*_ *, S*_*7*_ *, S*_*8*_ *, S*_*10*_ *, S*_*11*_ *, S*_*12*_	*S*_*2*_	*I*
*S*_*3*_	*S*_*2*_ *, S*_*3*_	*S*_*1*_ *, S*_*3*_ *, S*_*4*_ *, S*_*6*_ *, S*_*7*_ *, S*_*10*_ *, S*_*11*_	*S*_*3*_	*II*
*S*_*4*_	*S*_*2*_ *, S*_*3*_ *, S*_*4*_ *, S*_*5*_ *, S*_*6*_ *, S*_*12*_	*S*_*1*_ *, S*_*4*_	*S*_*4*_	*IV*
*S*_*5*_	*S*_*5*_	*S*_*1*_ *, S*_*4*_ *, S*_*5*_ *, S*_*10*_ *, S*_*11*_	*S*_*5*_	*I*
*S*_*6*_	*S*_*2*_ *, S*_*3*_ *, S*_*6*_	*S*_*1*_ *, S*_*4*_ *, S*_*6.*_	*S*_*6*_	*III*
*S*_*7*_	*S*_*2*_ *, S*_*3*_ *, S*_*7*_	*S*_*7*_ *, S*_*10*_ *, S*_*11*_	*S*_*7*_	*III*
*S*_*8*_	*S*_*2*_ *, S*_*8*_	*S*_*8*_ *, S*_*10*_ *, S*_*11*_	*S*_*8*_	*II*
*S*_*9*_	*S*_*9*_	*S*_*1*_ *, S*_*9*_ *, S*_*10*_ *, S*_*11*_	*S*_*9*_	*I*
*S*_*10*_	*S*_*2*_ *, S*_*3*_ *, S*_*5*_ *, S*_*7*_ *, S*_*8*_ *, S*_*9*_ *, S*_*10*_ *, S*_*12*_	*S*_*10*_ *, S*_*11*_	*S*_*10*_	*IV*
*S*_*11*_	*S*_*2*_ *, S*_*3*_ *, S*_*5*_ *, S*_*7*_ *, S*_*8*_ *, S*_*9*_ *, S*_*10*_ *, S*_*11*_ *, S*_*12*_	*S*_*11*_	*S*_*11*_	*V*
*S*_*12*_	*S*_*2*_ *, S*_*12*_	*S*_*1*_ *, S*_*4*_ *, S*_*10*_ *, S*_*11*_ *, S*_*12*_	*S*_*12*_	*II*

### 3.4. The ISM-based hierarchical framework

**Fig 3:**
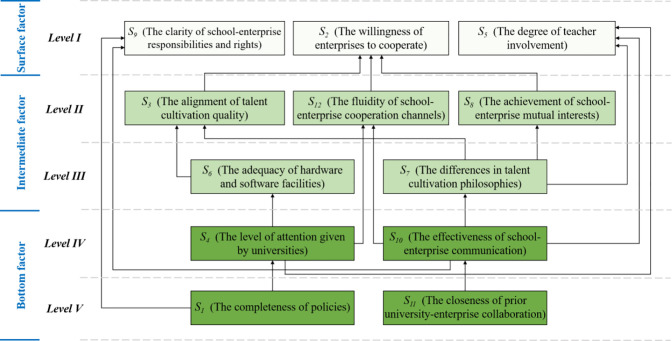
The ISM-based hierarchical framework.

As shown in Fig 3, the ISM-based hierarchical framework was established based on the mutual influencing relationship between the constraints and the level partition. In this framework, the constraints at *Level I* were called the surface factors, which were “S_2_ (The willingness of enterprises to cooperate),” “S_5_ (The degree of teacher involvement),” and “S_9_ (The clarity of school-enterprise responsibilities and rights)”; The 5 constraints were located at *Level II* and *Level III*, which were called the intermediate factors, i.e., “S_3_ (The alignment of talent cultivation quality),” “S_8_ (The achievement of school-enterprise mutual interests),” “S_12_ (The fluidity of school-enterprise cooperation channels),” “S_6_ (The adequacy of hardware and software facilities)” and “S_7_ (The differences in talent cultivation philosophies)”; The constraints at *Level IV* and *Level V* were called the bottom factors, which were “S_4_ (The level of attention given by universities),” “S_10_ (The effectiveness of school-enterprise communication),” “S_1_ (The completeness of policies)” and “S_11_ (The closeness of prior university-enterprise collaboration)”.

### 3.5. Constraining mechanisms

**Fig 4:**
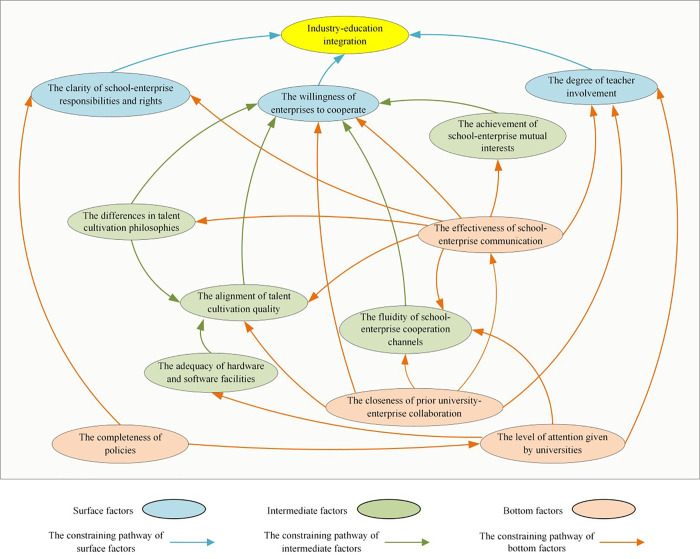
The constraint mechanism of IEI development in vocational education.

The mechanisms constraining the development of IEI in vocational education were identified through the ISM-based hierarchical framework and structural self-interaction matrix, as illustrated in Fig 4.

The results indicate that the ineffective implementation is primarily attributed to enterprises’ weak willingness to participate, ambiguous delineation of responsibilities, rights, and obligations between schools and enterprises during collaboration, and insufficient teacher engagement. These direct factors are further shaped by various intermediate- and bottom-level factors, which exert either direct or indirect influences. Specifically, the critical constraining factors include inadequate policies and regulations for IEI, a weak foundation for school-enterprise collaboration, insufficient institutional commitment from vocational colleges, and ineffective communication during implementation. These bottom-level factors directly or indirectly impact the adequacy of software/hardware facilities (e.g., teaching staff, training venues, equipment), the misalignment between educational philosophies and industry needs, the discrepancy between graduate competencies and employer requirements, the effectiveness of collaboration channels, and the fulfillment of mutual benefits and expectations. As intermediate-level factors, they further constrain development by decreasing enterprise willingness, obscuring clarity in responsibilities/rights/obligations, and undermining teacher motivation in the IEI process.

Through a systematic analysis of the interrelationships among constraining factors, 16 constraining pathways inhibiting the development of IEI in vocational education were identified, as detailed in Table 6. The root causes analysis reveals that two bottom-level factors – the completeness of policies and the strength of prior university-enterprise collaboration – predominantly influence other mediating factors, ultimately constraining the progress of IEI.

**Table 6:** Constraining pathways to IEI development in vocational education.

No.	Constraining pathway
1	The completeness of policies→The clarity of school-enterprise responsibilities and rights→IEI
2	The completeness of policies→The level of attention given by universities→The degree of teacher involvement→IEI
3	The completeness of policies→The level of attention given by universities→The fluidity of school-enterprise cooperation channels→The willingness of enterprises to cooperate→IEI
4	The completeness of policies→The level of attention given by universities→The adequacy of hardware and software facilities→The alignment of talent cultivation quality→The willingness of enterprises to cooperate→IEI
5	The closeness of prior university-enterprise collaboration→The degree of teacher involvement→IEI
6	The closeness of prior university-enterprise collaboration→The effectiveness of school-enterprise communication→The degree of teacher involvement→IEI
7	The closeness of prior university-enterprise collaboration→The willingness of enterprises to cooperate→IEI
8	The closeness of prior university-enterprise collaboration→The fluidity of school-enterprise cooperation channels→The willingness of enterprises to cooperate→IEI
9	The closeness of prior university-enterprise collaboration→The alignment of talent cultivation quality→The willingness of enterprises to cooperate→IEI
10	The closeness of prior university-enterprise collaboration→The effectiveness of school-enterprise communication→The willingness of enterprises to cooperate→IEI
11	The closeness of prior university-enterprise collaboration→The effectiveness of school-enterprise communication→The alignment of talent cultivation quality→The willingness of enterprises to cooperate→IEI
12	The closeness of prior university-enterprise collaboration→The effectiveness of school-enterprise communication→The differences in talent cultivation philosophies→The willingness of enterprises to cooperate→IEI
13	The closeness of prior university-enterprise collaboration→The effectiveness of school-enterprise communication→The fluidity of school-enterprise cooperation channels→The willingness of enterprises to cooperate→IEI
14	The closeness of prior university-enterprise collaboration→The effectiveness of school-enterprise communication→The achievement of school-enterprise mutual interests→The willingness of enterprises to cooperate→IEI
15	The closeness of prior university-enterprise collaboration→The effectiveness of school-enterprise communication→The differences in talent cultivation philosophies→The alignment of talent cultivation quality→The willingness of enterprises to cooperate→IEI
16	The closeness of prior university-enterprise collaboration→The effectiveness of school-enterprise communication→The clarity of school-enterprise responsibilities and rights→IEI

Regarding the completeness of policies, four distinct constraint pathways were established: (1) The existing incompleteness of policies governing IEI leads to unclear definitions of responsibilities, rights, and obligations between vocational institutions and enterprises; (2) These inadequacies significantly weaken institutional emphasis on integration implementation, consequently reducing faculty engagement; (3) Insufficient policy support adversely affects the establishment of effective school-enterprise collaboration channels; and (4) Policy gaps impede the development of essential supporting infrastructure and resources. Specifically, incomplete policies often lack clear funding provisions for vocational institutions to upgrade their training equipment or build industry-aligned training facilities. They also fail to set standards for corporate contributions of hardware, such as equipment donations or on-site training space. This leads to chronically underfunded hardware infrastructure. Notably, ineffective collaboration channels directly undermine corporate participation willingness, while inadequate infrastructure indirectly impacts enterprise engagement by weakening the alignment between institutional talent cultivation outcomes and industry workforce requirements. Collectively, both insufficient enterprise willingness and low faculty motivation directly hinder the effective implementation of IEI.

Regarding the closeness of prior university-enterprise collaboration, a total of 12 constraining pathways were identified. Among these, two pathways hinder the development of IEI in vocational education by diminishing teachers’ motivation for engagement. Specifically, the lack of a robust foundation for prior collaboration between universities and enterprises directly deminishes teachers’ motivation to participate in IEI. Additionally, it indirectly affects teachers’ enthusiasm by impairing the effectiveness of communication and interaction between universities and enterprises. Furthermore, nine pathways constrain the development of IEI by reducing enterprises’ willingness to collaborate. Specifically, a weak foundation of prior collaboration directly diminishes enterprises’ motivation to engage in IEI. It also indirectly lowers their willingness by reducing the smoothness of collaboration channels, the alignment between the quality of talent cultivation in universities and enterprises’ employment requirements, and the effectiveness of university-enterprise communication. Notably, the effectiveness of communication further indirectly influences enterprises’ willingness by directly affecting the differences in talent cultivation philosophies between universities and enterprises, as well as the fulfillment of mutual interests and expectations in IEI. Ultimately, these factors impede the implementation of IEI in vocational education. In addition, one pathway constrains the development of IEI by affecting the delineation of responsibilities, rights, and obligations between universities and enterprises. Specifically, a weak foundation of prior collaboration weakens the effectiveness of communication, and the lack of effective interaction hinders the clarification of each party’s responsibilities, rights, and obligations in the implementation process of IEI, thereby undermining its effective advancement.

## 4. Strategies for promoting IEI

To promote IEI development in vocational education, this study proposes targeted strategies for the government, institutions, and enterprises based on the identified constraint mechanisms.

### 4.1. Government: Strengthening institutional safeguards

The findings reveal that inadequate policies are a critical constraint on IEI. Therefore, the government should provide policy guidance and legal frameworks to standardize and promote its development. First, the responsibilities, rights, and obligations of vocational institutions and enterprises in IEI should be clearly defined to regulate their conduct while safeguarding their interests. Second, an evaluation and assessment mechanism should be established to enable comprehensive, process-based management, supervision, and evaluation of vocational institutions’ efforts in advancing IEI, thereby increasing institutional commitment. Third, the government should introduce incentive policies, such as rewards and tax benefits, by formulating a clear list of measures for enterprise participation to encourage deeper engagement in IEI.

### 4.2. Vocational institution: Strengthening the foundation of university-enterprise collaboration

The results indicate that weak prior collaboration between vocational institutions and enterprises is another major constraint. To facilitate healthy and sustainable development, vocational institutions should focus on establishing and consolidating early-stage partnerships to deepen and solidify collaboration. vocational institutions can strengthen university-enterprise collaboration through the following approaches: First, they should proactively seek partnerships with industry-leading enterprises, engaging in joint research and development, curriculum design, work-integrated learning (e.g., internships), and other collaborative activities. Second, vocational institutions and enterprises should jointly establish industry-academia-research innovation platforms to tackle key technological challenges, assisting enterprises in solving real-world technical problems. Third, vocational institutions should customize employee training and continuing education programs based on enterprise needs, enhancing workforce upskilling and supporting high-quality enterprise development.

### 4.3. Enterprise: Proactively assuming educational responsibilities

The findings also suggest that weak enterprise willingness to collaborate is a direct constraint, primarily due to unmet expectations regarding benefits. Thus, enterprises should move beyond a purely profit-driven mindset, recognize the long-term value of IEI, and actively contribute to educational development by fostering a corporate culture conducive to deeper collaboration. First, enterprises should engage in collaborative talent development, such as co-establishing modern industrial colleges with vocational institutions to cultivate high-quality applied talent. To address cost concerns that may hinder enterprise participation, a clear cost-sharing framework could be adopted: enterprises may primarily invest in industry-relevant equipment, on-site training venues, and part-time enterprise instructors, while vocational institutions take charge of curriculum design, full-time faculty allocation, and daily operation management. This division balances resource input and aligns with each party’s core strengths. Additionally, enterprises should leverage policy incentive support—such as applying for government subsidies for industry-education integration projects or tax deductions for educational investments—to offset partial costs, thereby enhancing the economic feasibility of joint modern industrial college establishment. They should also provide in-service training and continuous education to ensure employees’ skills remain aligned with industry advancements. Second, enterprises should contribute educational resources, such as instructors, equipment, and training bases, to enhance students’ practical skills while improving resource utilization efficiency. Third, enterprises should encourage employees to participate in vocational institution teaching and student mentoring, strengthening corporate social responsibility while enhancing employees’ professional and leadership competencies.

## 5. Conclusions

IEI is a critical strategic measure for advancing vocational education development. However, with the acceleration of industrial upgrading and technological transformation, the mismatch between traditional talent cultivation models and market demands has become increasingly prominent, necessitating an in-depth clarification of the constraints hindering IEI in vocational education. Therefore, this study takes Shenzhen, China, as an empirical case to systematically explore the constraints, mechanisms, and strategies for IEI in vocational education. The findings reveal 12 constraining factors, categorized into five hierarchical levels, forming 16 constraining pathways. Specifically, imperfect policies and weak preliminary industry-education collaboration are identified as deep-rooted factors. These influence intermediate factors such as institutional commitment and communication effectiveness, ultimately manifesting as surface-level issues, including insufficient corporate engagement and ambiguous rights-responsibility allocation between stakeholders. Notably, corporate willingness to participate is affected by nine distinct pathways, emerging as the core bottleneck impeding IEI implementation. In summary, this study’s primary contribution lies in its empirical identification of a multi-level constraint system for IEI, with corporate participation as the central barrier. A key innovation is the precise revelation of 16 influencing pathways, which systematically uncovers the underlying mechanisms—from policy and foundational collaboration to intermediate linkages and final outcomes—thereby providing a comprehensive theoretical basis for formulating targeted promotion strategies. To effectively promote IEI in vocational education, collaborative efforts from the government, vocational institutions, and enterprises are essential. The government should strengthen institutional safeguards, vocational institutions must consolidate the foundation for industry collaboration, and enterprises ought to actively fulfill their educational responsibilities.

However, this study has limitations. It focuses exclusively on Shenzhen, China, a global manufacturing hub and national educational reform pilot with unique industrial agglomeration, such as high-tech and advanced manufacturing, and policy support, which may restrict the generalizability of findings to regions with different economic structures or institutional environments. Additionally, it neglects sectoral variations in IEI: manufacturing, including electronics and automotive parts, relies on formalized, equipment-intensive IEI models, such as long-term internships, while construction prioritizes project-based, flexible collaborations, leading to heterogeneous impacts of identified constraints. For example, policy clarity is more critical for manufacturing, while communication effectiveness is more important for construction. Future research can address these limitations by: 1) expanding to cross-regional or cross-country comparisons, for instance, comparing Shenzhen with Guangzhou, or China with Germany’s dual-system, to test constraint mechanism generalizability; 2) conducting industry-specific studies, such as optimizing equipment-sharing policies for manufacturing, designing stable contracts for construction, and exploring skill mismatch in service sectors like healthcare; and 3) integrating mixed methods, including quantitative surveys across sectors and qualitative longitudinal enterprise cases, to refine constraint pathways, thereby providing a more context-aware understanding of IEI development.

## Supporting information

S1 Text Score statistics of potential constraining factors. (DOCX)

S2 Text Demographic information of the interviewees. (DOCX)
