# Supplementary material for: Constraints, mechanisms, and strategies for industry-education integration in vocational education: An empirical study
Source: PLoS One. 2025 Dec 18;20(12):e0339158. doi: 10.1371/journal.pone.0339158 (PMC12714252; doi:10.1371/journal.pone.0339158)
Supplement: S1 Text — (DOCX) [file pone.0339158.s001.docx]

**S1 Text.** Score statistics of potential constraining factors.

| No. | Potential constraining factors | Number of scores | | | | | Number of scores ≥4 | Percentage of scores ≥4 (%) | Constraining factor (Yes/No) |
| --- | --- | --- | --- | --- | --- | --- | --- | --- | --- |
|  |  | Score = 1 | Score = 2 | Score = 3 | Score = 4 | Score = 5 |  |  |  |
| 1 | The completeness of policies | 0 | 0 | 62 | 270 | 202 | 472 | 88.39 | Yes |
| 2 | The willingness of enterprises to cooperate | 0 | 0 | 22 | 253 | 259 | 512 | 95.88 | Yes |
| 3 | The alignment of talent cultivation quality | 0 | 1 | 25 | 226 | 282 | 508 | 95.13 | Yes |
| 4 | The level of attention given by universities | 0 | 4 | 55 | 292 | 183 | 475 | 88.95 | Yes |
| 5 | The degree of teacher involvement | 0 | 5 | 70 | 312 | 147 | 459 | 85.96 | Yes |
| 6 | The extent of student engagement | 13 | 95 | 247 | 162 | 17 | 179 | 33.52 | No |
| 7 | The adequacy of hardware and software facilities | 1 | 2 | 72 | 287 | 172 | 459 | 85.96 | Yes |
| 8 | The differences in talent cultivation philosophies | 0 | 0 | 41 | 217 | 276 | 493 | 92.32 | Yes |
| 9 | The achievement of school-enterprise mutual interests | 0 | 0 | 3 | 212 | 319 | 531 | 99.44 | Yes |
| 10 | The clarity of school-enterprise responsibilities and rights | 0 | 2 | 37 | 313 | 182 | 495 | 92.70 | Yes |
| 11 | The effectiveness of school-enterprise communication | 0 | 0 | 22 | 366 | 146 | 512 | 95.88 | Yes |
| 12 | The closeness of prior university-enterprise collaboration | 0 | 2 | 55 | 312 | 165 | 477 | 89.33 | Yes |
| 13 | The fluidity of school-enterprise cooperation channels | 0 | 4 | 39 | 328 | 163 | 491 | 91.95 | Yes |
