# Supplementary material for: Constraints, mechanisms, and strategies for industry-education integration in vocational education: An empirical study
Source: PLoS One. 2025 Dec 18;20(12):e0339158. doi: 10.1371/journal.pone.0339158 (PMC12714252; doi:10.1371/journal.pone.0339158)
Supplement: S2 Text — (DOCX) [file pone.0339158.s002.docx]

**S2 Text.** Demographic information of the interviewees.

| Variable | Category | Number | Percentage (%) |
| --- | --- | --- | --- |
| Gender | Male | 14 | 46.67 |
|  | Female | 16 | 53.33 |
| Age | Under 35 years old | 4 | 13.33 |
|  | 36-45 years old | 7 | 23.33 |
|  | 46-55 years old | 13 | 43.33 |
|  | Over 56 years old | 6 | 20 |
| Education background | Undergraduate | 7 | 23.33 |
|  | Postgraduate | 14 | 46.67 |
|  | Doctor | 9 | 30 |
| Occupation | Governmental staff | 6 | 20 |
|  | Scholar or researcher | 14 | 46.67 |
|  | Enterprise employees | 10 | 33.33 |
| Working experience | Less than 10 years | 5 | 16.67 |
|  | 11-20 years | 10 | 33.33 |
|  | 21-30 years | 11 | 36.67 |
|  | More than 31 years | 4 | 13.33 |
